# The London Practice of Medicine Contains a Cure, by Mr. Coote, of Inflammation and Abcess of the Tongue

**Published:** 1852-10

**Authors:** 


### ARTCLE XIV.

### *The London Practice of Medicine contains a Cure, by Mr. Coote, of Inflammation and Abscess of the Tongue.*

The tongue, he states, is an organ so frequently the seat of disease, that a few words respecting its structure may be interesting. The muscles, usually described as entering into its formation, are insufficient to account for the extent and variety of its movements; whilst mention is rarely made of those deeper and more central parts in which the products of inflammation are occasionally effused.

The mucous membrane is one of the most highly organized of the whole body: numerous papillæ, which, though commonly divided into three sets, pass insensibly one into the other, spring from every part of its dorsum. Under the mucous membrane of both the upper and lower surface lies a layer of transverse muscular fibres connected with the integument. Vertical muscular fibres, separated by wide intervals, extend from the upper to the lower layer, forming near the tip of the tongue a sort of open structure, the interspaces being occupied by an aggregation of vesicles, which, according to Gerlach, bear a close resemblance to salivary glands. "In these interspaces lie elongated glandular masses, consisting of numerous vesicles, which in size and structure, precisely correspond with those of the salivary glands. Each of these glandular bodies possesses its own excretory duct, which opens on the under surface of the tongue.

These are the glands discovered by Blandin, and recently brought into notice by Nuhn, who seems to think, from his investigations upon animals, that they are peculiar to man. The ducts open by the free border of the plica fimbriata, a slightly elevated line on either side of the frænum, indicating the last trace of the separated embryonic attachment of the tongue with the floor of the mouth.

From the foramen cœcum to the epiglottis, the mucous membrane is smooth and devoid of papillæ; and the structure of the tongue is occupied by numerous mucous glands, which feel like hard knots under the integument; they do not exist at the tip of the organ. In cases of rapid swelling of the tongue, the intermuscular glandular structures near the tip are the seat of the disease; and it must have been from the inflammation attacking only one set, and from the swelling being confined to one side of the organ, and encroaching especially upon its under part, that the unusual displacement occurred recorded by Wormald, who found that part of the under surface known as the plica fimbriata twisted towards the roof the mouth.

Abscesses sometimes form in these glands of Blandin. A lady, in good health, married, and the mother of a healthy family, consulted Mr. Stanley for a hard but painless tumor, the size of a small bean, imbedded in the substance of the tongue, near its tip. The mucous membrane covering it was raised, but loose and healthy. Upon its being grasped, there was an obscure sense of fluctuation communicated to the fingers, but, from a more superficial examination, it would have been pronounced solid. Although cancer almost invariably commences on the edge of the tongue, its origin being often referred to the irritation produced by the rough edge of a carious tooth, yet, the patient's age being considered, and the frequency with which malignant affections attack this organ, it was thought right to investigate by operation the character of a tumor which was slowly increasing in size, and to extirpate entirely, if it proved to be of questionable character. Chloroform having been administered, an incision was made into it with a lancet, when there escaped about half a teaspoonful of well-formed, healthy pus. There was a slight discharge for a few days, after which the wound closed, and the patient has had no further return of the complaint.

A working man, aged 40, married, and in good health, was received into the hospital, Dec. 6, 1850, under Mr. Stanley, for disease of the tongue. The whole of the anterior part of the organ was thickened and swelled; the mucous membrane was tense and smooth, and the surface was elevated and irregular. Two knots, about the size of large peas, which communicated an indistinct feeling of fluctuation when grasped between the finger and thumb, occupied the substance of the tongue. The patient had been married for many years, and had never had syphilis, but he had long been accustomed to walk about, while engaged at his work, with a short clay pipe in his mouth pressing against the tongue. Mr. Stanly punctured both the knots, and there escaped from each a small quantity of healthy matter. The patient left the hospital with directions, that he should, for the future, avoid exposing the organ to that which was obviously a source of irritation to it.

It must have been some rare affection of these glands which has been described by the late Mr. Earle:—"Clusters of very minute transparent vesicles pervaded the whole thickness of the tongue, occupying nearly one half, and projecting considerably both above and below that organ. The slightest injury caused them to bleed profusely, and in some places the clusters were separated by deep clefts, which discharged a fetid, irritating sanies. This disease which had resisted various plans of treatment, both local and constitutional, gradually yielded to perfect quiet, cleanliness, and large doses of hyoscyamus, which were increased to a drachm of the extract daily.

The disease with which suppuration in the glandular structure of the tongue is most likely to be confounded is syphilitic thickening; both affections commence near the mesial line in the substance of the organ, and the thickness of the walls of the cyst in the former, renders it to the feel the same as a solid tumor. In syphilis it happens generally, but not always, that other symptoms are present at the same time, such as superficial ulceration of the tonsils and fauces, or some eruption of the skin; but when the induration occurs alone, and at a considerable time from the occurrence of the primary sore, it often happens that the diagnosis is attended with considerable difficulty. A gentleman consulted Mr. Lawrence with a thickened and elevated state of the middle of the dorsum of the tongue; the indurated knot was equal in size to a sixpence, and was covered by smooth and red mucous membrane. Upon being closely questioned, he stated that eight months previously he had had an excoriation of the prepuce, which had appeared two months after connexion, and had lasted only four days. Further examination detected superficial ulceration of the mucous membrane of the velum and fauces, which, he acknowledged, had rendered deglutition difficult; but, as it had existed nearly six months, he had ceased to think much about it, attributing it to disorder of the stomach. He had been to the seaside, and had adopted various measures for the improvement of his general health, but the state of the throat remained the same. Under a mild mercurial course he recovered completely. A young woman, aged 26, was under Mr. Lawrence's care in the hospital, with a syphilitic affection of the tongue as the only symptom. She was married and in respectable circumstance: she had never, to her knowledge, had primary venereal disease, but three or four years ago she had had an eruption, described as scaly, and a sore throat, both of which lasted for some weeks. The disease in the tongue yielded to a mild course of mercury.

